# Role of MMP-1 (-519A/G, -1607 1G/2G), MMP-3 (Lys45Glu), MMP-7 (-181A/G), and MMP-12 (-82A/G) Variants and Plasma MMP Levels on Obesity-Related Phenotypes and Microvascular Reactivity in a Tunisian Population

**DOI:** 10.1155/2017/6198526

**Published:** 2017-11-26

**Authors:** Soumaya Boumiza, Sarra Bchir, Hela ben Nasr, Ammar Abbassi, Marie-Paule Jacob, Xavier Norel, Zouhair Tabka, Karim Chahed

**Affiliations:** ^1^Unité de recherche UR12ES06, Physiologie de l'Exercice et Physiopathologie: de l'Intégré au Moléculaire “Biologie, Médecine et Santé,” Faculté de Médecine de Sousse, Université de Sousse, Sousse, Tunisia; ^2^Faculté des Sciences de Bizerte, Université de Carthage, Bizerte, Tunisia; ^3^Institut des Sciences Infirmières, Sousse, Tunisia; ^4^District Medical du Centre, CNAM, Sousse, Tunisia; ^5^Laboratory for Vascular Translational Science (LVTS), INSERM U1148, CHU X. Bichat, 46 rue Henri Huchard, 75877 Paris Cedex 18, France; ^6^Faculté des Sciences de Sfax, Université de Sfax, Sfax, Tunisia

## Abstract

**Aims:**

The impact of MMP-1 (-519A/G, -1607 1G/2G), MMP-3 Lys45Glu (A/G), MMP-7 -181A/G, and MMP-12 -82A/G variants and plasma MMP levels on obesity and microvascular reactivity in Tunisians.

**Methods:**

Our population included 202 nonobese and 168 obese subjects. Anthropometric, biochemical, and microvascular parameters were determined according to standard protocols. PCR-RFLP and ELISA were used to determine the genetic variants and levels of MMPs, respectively.

**Results:**

The MMP-3 45Glu (G) allele associates with higher anthropometric values and MMP-3 levels compared to AA genotype carriers (BMI (kg/m^2^): 30 ± 0.51 versus 27.33 ± 0.8, *P* = 0.004; MMP-3 levels: 7.45 (4.77–11.91) versus 5.21 (3.60–10.21) ng/ml, *P* = 0.006). The MMP-12 -82G allele was also associated with higher BMI values when compared to subjects carrying the AA genotype (31.41 ± 0.85 versus 28.76 ± 0.43, *P* < 0.001). Individuals carrying the MMP-3 45G or MMP-12 -82G variants were also associated with a higher risk for severe forms of obesity (MMP-3: OR = 1.9, *P* = 0.002; MMP-12: OR = 2.63, *P* = 0.003). Similarly, the MMP-7 -181G allele was associated with a higher MMP-7 level and an increased risk for morbid obesity when compared to AA genotype carriers (0.32 (0.31–0.60) versus 0.18 (0.17–0.24) ng/ml, *P* = 0.01; OR = 1.67, *P* = 0.02, resp.).

**Conclusion:**

MMP-3, MMP-7, and MMP-12 polymorphisms associate with obesity risk and its severity.

## 1. Introduction

Obesity is a pathological condition that is closely related to genes with environmental modulators, including sedentary life and positive energy balance [1]. It is characterized by chronic inflammation and secretion of adipokines and matrix metalloproteinases (MMPs) [2]. According to the literature, MMPs contribute to adipose tissue remodeling through the degradation of extracellular matrix (ECM) components and control of adipogenesis [3]. Among these, MMP-1 has been shown to degrade fibrillar collagens while MMP-3 has a wide range of ECM substrates such as nonfibrillar collagens and laminin [4]. MMP-7 is another metalloproteinase that has a broad substrate specificity for ECM proteins including gelatin and type IV collagen, while MMP-12 is a key macrophage-derived enzyme that has a role in adipose tissue remodeling [5, 6].

Until now, SNPs in MMP genes have been described to alter their expression [7]. As an example, the MMP-1 (-1607 1G/2G, rs1799750) variant creates an Ets-binding site at position (-1607) which increases the expression of the corresponding gene [8]. The -519A/G (rs1144393) SNP consists in the substitution of guanine to adenine, but its role in the expression of the MMP-1 gene is unclear. The MMP-3 Lys45Glu (A/G) (rs679620) encodes a Lys-Glu nonsynonymous variant resulting in a putative functional modification of MMP-3 [9]. The -181A/G variant in MMP-7 (rs11568818) has also been reported to affect the binding of various nuclear proteins leading to an increase of transcriptional activity [10]. The -82A/G SNP of MMP-12 (rs2276109) has been shown to affect an AP-1-binding site and modulates MMP-12 expression [11].

Up to now, only a few studies have determined the impact of MMP variants and plasma MMP levels on obesity-related phenotypes and microvascular reactivity. The goals of the current report were to explore the impact of MMP-1 (-519A/G; -1607 1G/2G), MMP-3 Lys45Glu (A/G), MMP-7 -181A/G, and MMP-12 -82A/G SNPs on the development of obesity and its severity among Tunisians. We also determined whether these SNPs affect biochemical and microvascular reactivity, as well as plasma levels of the corresponding proteins.

## 2. Materials and Methods

### 2.1. Study Population

370 randomly selected Tunisian subjects (181 men and 189 women) with a mean age of 38 years were selected for the current study. 202 individuals were classified as nonobese subjects (18.5 ≤ BMI < 25 kg/m^2^) while 168 subjects were obese (BMI ≥ 30 kg/m^2^). Obese individuals were categorized according to the WHO classification as class I with 30 ≤ BMI < 35 kg/m^2^, class II with 35 ≤ BMI < 40 kg/m^2^, and class III (morbidly obese) with 40 kg/m^2^ ≤ BMI [12]. Overweight subjects defined as 25 ≤ BMI < 30 kg/m^2^ were excluded from the study. Diabetic individuals and subjects with a history of cardiovascular disease, smoking habits, malignancy, and renal, thyroid, and liver diseases were also excluded from the study. Other exclusion criteria were subjects using medications that might alter the endothelial or smooth muscle-dependent responses. All participants signed a written informed consent, and the study was approved by the Farhat Hached Hospital Ethics Committee.

### 2.2. Anthropometric and Biochemical Analyses

For all participants, weight (kg), height (m), BMI (kg/m^2^), waist circumference (WC) (cm), and hip circumference (HC) (cm) were determined according to standard protocols. Waist-to-height ratio (WHtR) and waist-to-hip ratio (WHR) were also measured by WC/height ratio and WC/HC ratio, respectively. The fasting glucose (FG), total cholesterol (TC), and triglycerides (TG) were determined by the corresponding oxidase methods (Elitech Diagnostic, France). The immune-inhibition method (Elitech Diagnostic, France) was used to measure high-density lipoprotein cholesterol concentration (HDL-C) while LDL-C was determined with the Friedwald formula [13]. The sensitive immunonephelometric method was used to measure the apolipoprotein (apo) A1, (apo) B, and high-sensitivity C-reactive protein (hs-CRP) (BNII, Dade Behring, Marburg, Germany).

### 2.3. Microvascular Reactivity Assessments

Endothelial function was determined in all subjects by assessing the forearm microvascular cutaneous vasoreactivity using laser Doppler flowmetry coupled with iontophoresis (Periflux PF5000; Perimed, Stockholm, Sweden) [14]. The maximum forearm skin blood flow (FSBF_max_) represented the endothelium-dependent response, registered after three cumulative doses of acetylcholine (ACh) (arbitrary unit). The forearm flow response to heating the skin (FSBF_RHS_) represented the endothelium-independent response registered after the increase in skin temperature (44°C) without ACh infusion (arbitrary unit). Data were also presented as cutaneous vascular conductance (CVC) that represents the cutaneous blood flow (CBF)/mean arterial pressure (MAP) ratio. The MAP was determined as 1/3 SBP + 2/3 DBP. The difference between the peak CVC upon ACh stimulation (after three doses of ACh) and the baseline CVC was considered as the endothelium-dependent response (ΔACh-CVC). The difference between the peak CVC following RHS-induced vasodilation and the baseline was considered as the endothelium-independent response (ΔRHS-CVC) [15].

### 2.4. Genotyping of MMP-1 (-519A/G; -1607 1G/2G), MMP-3 Lys45Glu (A/G), MMP-7 -181A/G, and MMP-12 -82A/G Variants

**Figure 1:**
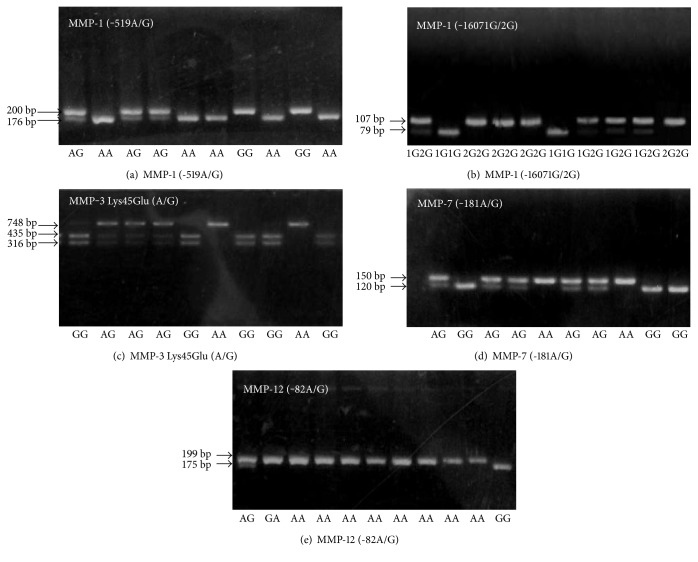
Genotyping of MMP-1 (-519A/G; -1607 1G/2G), MMP-3 Lys45Glu (A/G), MMP-7 (-181A/G), and MMP-12 (-82A/G) by PCR-RFLP. (a) MMP-1 (-519A/G): the product (200 bp) was digested with KpnI. The A allele corresponds to the 176 bp and 24 bp fragments. The G allele corresponds to the 200 bp. (b) MMP-1 (-1607 1G/2G): the PCR product (118 bp) was digested with XmnI. The 1G allele corresponds to the 79 bp, 28 bp, and 11 bp. The 2G allele corresponds to the 107 bp and 11 bp fragments. (c) MMP-3 Lys45Glu (A/G): the PCR product (748 bp) was digested with TaqI. The G allele corresponds to the 432 bp and 316 bp fragments. The A allele corresponds to the 748 bp fragment. (d) MMP-7 (-181A/G): the PCR product (150 bp) was digested with EcoRI. The A allele corresponds to the 150 bp fragment. The G allele corresponds to the120 bp and 30 bp fragments. (e) MMP-12 (-82A/G): the PCR product (199 bp) was digested with PvuII. The A allele corresponds to the 199 bp fragment. The G allele corresponds to the 175 bp and 24 bp fragments.

DNA was isolated as previously described [16]. Allelic discrimination between variants was performed by PCR-RFLP. The PCR reactions (25 *μ*l) contained 100 ng of genomic DNA, 25 *μ*M of each deoxynucleotide triphosphate (dNTP, Biotools Inc.), 50 pmol of each primer, 2.5 mM of MgCl_2_, and 0.5 units of Taq polymerase (Promega, Madison, WI, USA). Details of primers and PCR-RFLP conditions are reported in Table 1. The PCR digestions were repeated in more than 10% of the samples, to assess the reliability of genotyping, and no differences were found (Figure 1).

### 2.5. ELISA Analysis

MMP-1 and MMP-3 levels were determined by enzyme-linked immunosorbent assays (ELISA) using commercially available kits (DUOSET® ELISA, R&D Systems; DYS901 and DY513, resp.). MMP-7 and MMP-12 plasma levels were evaluated by Magnetic Luminex (R&D Systems Inc., USA & Canada; LMPM907 and LMPM919, resp.).

### 2.6. Statistical Analysis

SPSS® 17.0 software (Chicago, IL, USA) and GraphPad Prism (version 6.04) were used for statistical analyses. Comparisons of quantitative variables were carried out using a *t*-test or the Mann–Whitney *U* test as appropriate. Comparison of categorical variables including calculation for deviation from the Hardy–Weinberg equilibrium (HWE) was performed using the chi-square (*χ*^2^) test. The evaluation of odds ratios (ORs) and 95% confidence intervals (CIs) was carried out using an unconditional logistic regression model adjusted for age and sex. Haplotypes and linkage disequilibrium (LD) were analyzed with SNPstats (http://bioinfo.iconcologia.net/SNPstats). Results were considered statistically significant if the *P* value <0.05. The Bonferroni correction (*P*c) was applied to adjust for multiple comparisons.

## 3. Results

### 3.1. Anthropometric, Biochemical, and Microvascular Parameters of the Study Subjects

Anthropometric, biochemical, and microvascular values are indicated in Table 2. The subjects had a mean BMI value of 30 kg/m^2^ ± 0.44. In this study, we used a BMI cutoff point of 30 kg/m^2^ for obesity status. As expected, higher BMI and abdominal obesity values (WC, WHR, and WHtR) were associated with obesity status. SBP, DBP, MAP, and hs-CRP were increased in obese subjects. As indicated in Table 2, lipidic parameters (TG, TC, and LDL-C) were significantly increased among obese individuals, while HDL-C was decreased when compared to nonobese subjects. The association of microvascular endothelial function with obesity status was also performed. The FSBF_max_ as well as the FSBF dose-response to ACh expressed as area under the curve (AUC) was decreased in the obese group (*P* < 0.001). Basal CVC, as well as peak ACh-CVC and ΔACh-CVC values, was also decreased in obese subjects (*P* < 0.001). BMI and central adiposity (WC and WHR) were inversely correlated with the FSBF_max_ response to ACh (*r* = −0.45, *P* < 0.001; *r* − 0.40, *P* < 0.001; *r* = −0.33, *P* = 0.002, resp.). Additionally, basal CVC and peak ACh-CVC were inversely associated with BMI (*r* = −0.45, *P* = 0.002; *r* = −0.45, *P* = 0.002, resp.) and WC values (*r* = −0.40, *P* = 0.003; *r* = −0.26, *P* = 0.02, resp.). The abdominal obesity marker (WHtR) was inversely associated with peak ACh-CVC (*r* = 0.48, *P* = 0.001).

### 3.2. Impact of MMP-1, MMP-3, MMP-7, and MMP-12 Variants on Anthropometric Parameters in the Sampled Population

**Figure 2:**
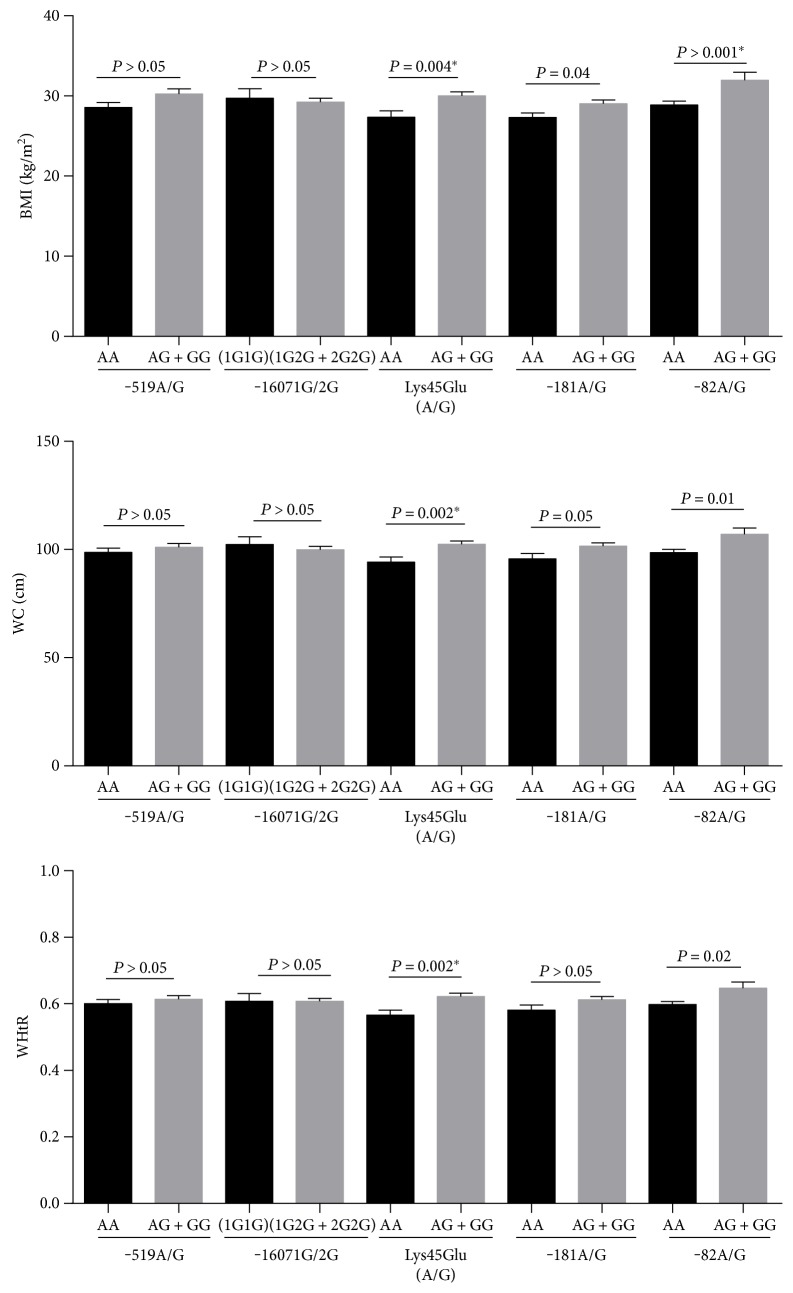
Variation of anthropometric parameters (BMI, WC, WHR, and WHtR) according to MMP-1 (-519A/G), MMP-1 (-1607 1G/2G), MMP-3 Lys45Glu (A/G), MMP-7 (-181A/G), and MMP-12 (-82A/G) polymorphisms in study subjects. Data are expressed as mean ± SD. Associations were analyzed using a dominant model. ^∗^*P* value remained significant after the Bonferroni correction. BMI: body mass index; WC: waist circumference; WHtR: waist-to-height ratio.

For the MMP-1 (-519A/G) polymorphism, no significant variation in BMI values was observed in individuals carrying the dominant model (AG+GG) genotype in comparison to AA genotype carriers (30.25 kg/m^2^ ± 0.61 versus 28.5 kg/m^2^ ± 0.63, Figure 2). Additionally, individuals carrying the AG or GG genotypes showed no significant association with abdominal obesity marker (WC, WHtR) values in comparison to AA genotype carriers (Figure 2). A similar result was observed for the MMP-1 (-1607 1G/2G) SNP in subjects carrying the 1G2G or 2G2G genotypes compared to 1G1G genotype carriers (Figure 2).

Regarding the MMP-3 Lys45Glu (A/G) SNP, individuals carrying the (AG/GG) genotype showed increased BMI, WC, and WHtR values when compared to AA genotype carriers (BMI (30 kg/m^2^ ± 0.51 versus 27.33 kg/m^2^ ± 0.8, *P* = 0.004); WC (102.45 cm ± 1.48 versus 94.1 cm ± 2.41, *P* = 0.002); WHtR (0.61 ± 0.01 versus 0.54 ± 0.01, *P* = 0.002), Figure 2). Investigation of the MMP-7 (-181A/G) polymorphism revealed a borderline association with BMI values. Subjects carrying the (AG/GG) genotype showed increased BMI values when compared to AA genotype carriers (BMI (29.16 kg/m^2^ ± 0.49 versus 27.19 kg/m^2^ ± 0.49, *P* = 0.04) (Figure 2)). Higher BMI, WC, and WHtR values were found among individuals carrying the MMP-12 -82 AG/GG genotype when compared to AA genotype carriers (BMI (31.41 kg/m^2^ ± 0.85 versus 28.76 kg/m^2^ ± 0.43, *P* < 0.001); WC (105.94 cm ± 2.43 versus 98.51 cm ± 1.22, *P* = 0.01); WHtR (0.64 ± 0.01 versus 0.59 ± 0.007, *P* = 0.02), Figure 2).

### 3.3. Impact of MMP-1, MMP-3, MMP-7, and MMP-12 Variants on Clinical Parameters, Microvascular Reactivity, and MMP Levels

The SBP, DBP, and MAP revealed no significant association with MMP-1, MMP-3, and MMP-7 polymorphisms. Regarding the MMP-12 (-82A/G) SNP, there was an increase in DBP among subjects carrying the AG or GG genotypes compared to AA genotype carriers (81 mmHg ± 1.2 versus 78.7 mmHg ± 0.54, *P* = 0.06). No significant correlation was observed between MMP-1, MMP-3, MMP-7, and MMP-12 polymorphisms, FG, and hs-CRP levels. No significant association was also found between MMP-1 and MMP-7 polymorphisms and TG, TC, LDL-C, and HDL-C levels. Regarding the MMP-3 polymorphism, a higher amount of LDL-C was found in subjects carrying the AG or GG genotypes compared to AA genotype carriers (2.97 mmol/l ± 0.07 versus 2.71 mmol/l ± 0.1, *P* = 0.04). A significant increase in LDL-C was also found among obese subjects carrying the AG or GG genotypes when compared to AA genotype carriers (3.25 mmol/l ± 0.1 versus 2.68 mmol/l ± 0.1, *P* = 0.01, *P*_c_ = 0.04). Investigation of the MMP-12 (-82A/G) SNP also revealed that individuals carrying the AG or GG genotypes present higher LDL-C levels when compared to AA genotype carriers (3.16 mmol/l ± 0.15 versus 2.84 mmol/l ± 0.06, *P* = 0.05). Analysis of endothelial function in the sampled population revealed, however, no significant relationships between the MMP-1, MMP-7, MMP-3, and MMP-12 variants and microvascular reactivity parameters (data not shown).

We also determined whether the MMP polymorphisms affect plasma levels of the corresponding proteins. For the MMP-1 (-519A/G, -1607 1G/2G) SNPs, there was no significant impact on MMP-1 levels in the sampled population (-519A/G, AA: 1.42 (0.56–3.42) ng/ml, AG+GG: 2 (0.75–4.16) ng/ml; –1607 1G/2G), 1G1G: 2.53 (0.75–4.17) ng/ml, 1G2G+2G2G: 1.66 (0.66–3.81) ng/ml). For the MMP-3 Lys45Glu (A/G) polymorphism, subjects carrying the AG or GG genotypes present higher MMP-3 levels compared to individuals having the AA genotype (7.45 (4.77–11.91) ng/ml versus 5.21 (3.6–10.21) ng/ml, *P* = 0.006, *P*_c_ = 0.024). When subjects were categorized according to severity of obesity, a higher level of MMP-7 was observed in morbidly obese subjects carrying the -181G variant (AG+GG: 0.32 (0.31–0.6), AA: 0.18 (0.17–0.24), *P* = 0.01, *P*_c_ = 0.04).

### 3.4. Distribution of MMP-1, MMP-3, MMP-7, and MMP-12 Variants According to Obesity Status and Severity

Analyses were carried out to study the distribution of the different SNPs according to obesity status and severity based on BMI (Tables 3 and 4). For all variants, the genotype distribution did not differ from that expected from the Hardy–Weinberg equilibrium. As indicated in Table 3, the frequency distribution of MMP-1 (-519) AA, AG, and GG genotypes in obese and nonobese subjects was 39.6%, 51.8%, and 8.6% and 53%, 41.5%, and 5.5%, respectively. The frequencies of A and G alleles in obese and nonobese subjects were 65.55% and 34.45% and 73.75% and 26.25%, respectively. Individuals carrying the -519 AG and AG+GG genotypes revealed a borderline association with obesity development when compared to the homozygous AA genotype (OR = 1.57, *P* = 0.03, *P*_c_ = 0.24; OR = 1.61, *P* = 0.02, *P*_c_ = 0.08, resp., Table 3). When subjects were categorized according to severity of obesity, we found no association in genotype and allele distributions between nonmorbidly obese, morbidly obese, and nonobese subjects (Table 4). For the MMP-1(-1607 1G/2G) SNP, the frequencies of 1G1G, 1G2G, and 2G2G genotypes in obese and nonobese subjects were 13.4%, 37.8%, and 48.8% and 12%, 37.7%, and 50.3%, respectively (Table 3). The frequencies of 1G and 2G alleles in obese and nonobese subjects were 32.32% and 67.68% and 30.9% and 69.1%, respectively. The -1607 (2G) allele revealed no significant association with obesity compared to the homozygous 1G allele (Table 3). When subjects were categorized according to severity of obesity, our results showed that the 1G2G as well as the 2G2G genotypes have no significant impact on nonmorbid and morbid obesity (Table 4). For the MMP-3 Lys45Glu (A/G) polymorphism, the frequencies of AA, AG, and GG genotypes were 20.1%, 56.7%, and 23.2% in obese and 34.2%, 49.5%, and 16.3% in nonobese subjects. Interestingly, the AG, GG, and the combined AG+GG genotypes were associated with obesity status compared to AA genotype carriers (OR = 1.92, *P* = 0.01; OR = 2.4, *P* = 0.006; OR = 2.04, *P* = 0.004, resp., Table 3). In addition, the G allele was associated with obesity development compared to A allele carriers, (OR = 1.52, *P* = 0.004, *P*_c_ = 0.016). When subjects were categorized according to severity of obesity, the frequency of the Lys45Glu (G) allele was more frequent in nonmorbidly obese (48.58%) compared to nonobese subjects (*P* = 0.07, Table 4). Interestingly, the frequencies of AG, GG, and AG+GG genotypes were more abundant in morbidly obese compared to nonobese individuals (OR = 2.83, *P* = 0.01; OR = 4.93, *P* = 0.002; OR = 3.31, *P* = 0.005, resp.) (Table 4). Besides, the G allele was associated with severe forms of obesity when compared to the A allele (OR = 1.9, *P* = 0.002, *P*_c_ = 0.008, Table 4). For the MMP-7 -181A/G SNP, the frequencies of AA, AG, and GG genotypes in obese and nonobese subjects were 23%, 55.2%, and 21.8% and 26.2%, 51%, and 22.8%, respectively. The -181G variant revealed no significant association with obesity development compared to the homozygous AA genotype (Table 3). When subjects were categorized according to severity of obesity, the frequencies of MMP-7 -181G variant were higher in morbidly obese subjects. The AG, GG, and the combined AG+GG genotypes were significantly associated with risk for morbid obesity when compared to AA genotype carriers (OR = 3.68, *P* = 0.01; OR = 3.4, *P* = 0.02; OR = 3.58, *P* = 0.008). For the MMP-12 (-82A/G) SNP, the frequencies of AA, AG, and GG genotypes were 76.4%, 23%, and 0.6% in obese participants and 88.6%, 11.4%, and 0% in nonobese participants. We found a significant association between the AG genotype, the combined genotypes AG+GG, and the development of obesity (OR = 2.33, *P* = 0.003; OR = 2.39, *P* = 0.002, resp.). The frequencies of A and G alleles were 94.28% and 5.72% in nonobese subjects and 87.88% and 12.12% in obese individuals. The G allele was associated with enhanced obesity when compared to A allele carriers (OR = 2.27, *P* = 0.002, Table 3). These associations remained significant after the Bonferroni correction. When subjects were categorized according to the severity of obesity, similar findings were found for both nonmorbidly and morbidly obese subjects in comparison to nonobese subjects. As indicated in Table 4, the AG genotype was associated with a higher risk for nonmorbid obesity when compared to AA genotype carriers (OR = 1.58, *P* = 0.01). The G allele was also associated with nonmorbid obesity when compared to the A allele (OR = 2.17, *P* = 0.008). Similarly, the AG genotype alone or in combination with the GG variant was associated with a higher risk for severe forms of obesity when compared to AA genotype carriers (OR = 2.2, *P* = 0.01; OR = 2.6, *P* = 0.01). The G allele was also significantly associated with morbid obesity when compared to the A allele (OR = 2.63, *P* = 0.003).

We further determined the haplotype frequencies of the different polymorphisms in obese and nonobese subjects. Linkage disequilibrium analysis revealed that MMP-3 and MMP-12 SNPs were in mild linkage disequilibrium (*D*′ = 0.42, *r* = 0.13). Haplotypes follow this order: MMP-1 (-1607 1G/2G), MMP-1 (-519A/G), MMP-7 (-181A/G), MMP-3 Lys45Glu (A/G), MMP-12 (-82A/G). Comparison of the haplotype frequencies between obese and nonobese groups revealed an increased risk for obesity for the haplotype s1G_−1607_:G_−519_:G_−181_:G_Lys45Glu_:A_−82_ (Supplementary File 1 available online at https://doi.org/10.1155/2017/6198526).

## 4. Discussion

Obesity is a heterogeneous disorder that is related to environmental and genetic factors. This phenotype is associated with the emergence of type 2 diabetes, hypertension, and cardiovascular disease [17]. As a proinflammatory state, obesity correlates also with the secretion of key regulators of adipocyte differentiation and energy balance such as adipokines and matrix metalloproteinases (MMPs) [18]. Among MMPs, MMP-1, MMP-3, MMP-7, and MMP-12 genes have been found to affect adipose tissue remodeling and obesity-related metabolic traits [19]. Genetic variations in MMP genes have also been reported as key regulators of MMP protein expression in several diseases [20, 21]. Up to now, only a few studies have determined, however, the link between SNPs in MMP genes and the emergence of obesity. The aims of the current study were, therefore, to assess the impact of MMP-1-519A/G (rs1144393), MMP-1 -1607 1G/2G (rs1799750), MMP-3Lys45Glu (A/G) (rs679620), MMP-7 -181A/G (rs11568818), and MMP-12 -82A/G (rs2276109) polymorphisms on obesity status and its anthropometric indicators. Also, we evaluated whether these variants are related to obesity-related phenotypes and explored their role on microvascular parameters and MMPs levels.

MMP-1 is a collagen-degrading enzyme that has an impact on ECM remodeling in both normal and pathological conditions. It is able to cleave different types of collagen (I, II, and III). Several studies reported an enhanced expression of MMP-1 in preadipocytes from obese subjects suggesting a role in ECM remodeling in obesity [22]. MMP-1 has also been found in preadipocytes/stromal vascular cells of human adipose tissue and its levels were increased in class II-III obese [23]. In the current study, no significant relationships were found between MMP-1 (-16071G/2G, -519A/G) variants and the anthropometric parameters investigated including BMI, WC, and WHtR, which may suggest that these SNPs do not affect body fat accumulation in the sampled population. We also determined the role of these SNPs on microvascular and metabolic parameters of the study subjects but no significant impact was observed. A similar result was found when subjects were categorized according to obesity status. Obese subjects were further stratified by BMI into classes I, II, and III but no significant relationships were observed with the different variants. We also determined the role of -1607 1G/2G and -519A/G variants on MMP-1 levels in the sampled population. According to the literature, the (2G) insertion variant at position -1607 creates an Ets-binding site (5′-GGA-3′) that increases the expression of MMP-1 gene [8]. Besides, Hsieh et al. [24] found that MMP-1 activity was significantly increased in serum from patients with 1G1G and 1G2G genotypes compared with 2G2G genotype carriers. The -519A/G SNP was significantly associated with myocardial infarction in British and Swedish individuals [25]. In the present report, we failed to find a significant impact of these polymorphisms on MMP-1 levels in the sampled population, as well as in subgroups of nonobese and obese subjects. These results contrast with data from Huang et al. [26] showing that the relationship between the -1607 1G/2G SNP and MMP-1 levels in a Taiwanese population was dependent on obesity status and that obese subjects carrying the 2G/2G genotype had lower MMP-1 levels compared to 1G/1G-carrying individuals. In line with the current findings, these authors revealed, however, no significant impact of the -519A/G variant on obesity status in Taiwan.

MMP-3 (stromelysin I) is a proteoglycanase and a key player in ECM degradation and remodeling [4]. MMP-3 was also associated with the inflammatory response during adipose tissue expansion, as well as with higher fat tissue levels, indicating a role of MMP-3 in adipogenesis [27]. According to the literature, alterations of MMP-3 pathway in obesity could be related to functional SNPs in MMP-3 gene. Nowadays, its common variants have received great interest in relation to cardiovascular diseases and hypertension [28, 29]. No studies have explored, however, the impact of MMP-3 Lys45Glu variant on obesity status. In the current study, we, therefore, determined the relationships between the MMP-3 Lys45Glu polymorphism and both anthropometric, microvascular, and MMP-3 levels in a Tunisian population. This polymorphism predicts an amino acid substitution (Lys45 to Glu) that is involved in maintaining MMP-3 latency [30]. In the current study, no significant impact of MMP-3 polymorphism was observed on microvascular reactivity parameters. Interestingly, higher values of anthropometric markers (BMI, WC, and WHtR) were associated with the Lys45Glu (A/G) variant in the sampled population, consolidating its role as a biomarker of obesity. These results corroborate those of Taylor et al. [9] revealing a significant relationship between the rs679620 and both BMI and WC in African-Americans. In contrast to our findings, Traurig and colleagues [31] reported that this SNP was not correlated with BMI values. Stratified analysis, based on obesity status, also revealed a significant association between the Lys45Glu (A/G) variant and body fat accumulation, which reflects that the 45G allele is a risk factor for obesity among Tunisians. When obese subjects were further stratified by BMI, significant relationships with the 45Glu variant were observed when morbidly obese subjects were considered. Additionally, we found a higher level of MMP-3 in 45G allele carriers in the studied population. A similar finding was obtained in both obese and morbidly obese subjects, suggesting that MMP-3 Lys45Glu variant could affect MMP-3 protein expression and is therefore related to obesity development and severity. According to the literature, MMP-3 could also be related to low-grade chronic inflammation in obesity. In line with this, Nakai et al. [32] found that CRP, which is a low-grade systemic inflammation marker, induced MMP-1, MMP-3, and MMP-9 expression in adipocyte-differentiated 3T3-L1 cells. Our study provides, however, no significant association between the investigated polymorphisms and hs-CRP levels. The dissociation of CRP and MMP levels may also highlight that hs-CRP has a marginal effect on serum levels of MMP-1, MMP-3, MMP-7, and MMP-12 in our Tunisian cohort study. These results corroborate those of Preston et al. [33] showing no significant correlation between systemic CRP and MMP levels in conditions of chronic inflammation. Although other studies are required, our observations on the expression of MMPs (e.g., MMP-3) could be a reflection of gene stimulation depending on other proinflammatory stimuli such as leptin and IL-1 [34]. Up to now, the specific role played by systemic MMPs in remodeling and expansion of adipose tissue has not been fully determined. In this regard, previous studies reported that increased serum levels of MMP-2 and MMP-9 in obese patients are not related to adipose tissue expansion and body mass index [35–37]. Interestingly, by using protein arrays, Kurki et al. [38] found increased levels of MMP-3 in tissue samples from visceral fat of obese mice. MMP-3 has also been reported to cleave osteopontin (OPN), a major regulator of adipocyte function [39]. Recently, it has also been found that MMP cleavage of OPN in adipose tissue enhances inflammatory and prodiabetic activities on adipocytes. From our results, the site of derivation of MMP-3 and whether it could be a useful indicator of an inflammatory process occurring in adipose tissue require further investigation.

MMP-7 (matrilysin-1) is another metalloproteinase with an inflammatory regulatory role that displays a proteolytic activity against various kinds of ECM components [40]. Previous studies demonstrated that decreased levels of MMP-7 may be considered as a marker of obesity [41]. Other reports demonstrated that MMP-7 levels positively correlate with fat diameter, as well as with markers of central obesity and obesity-related metabolic traits [42]. Alterations of MMP-7 levels in obesity could also be related to genetic variations in MMP-7 gene. According to the literature, the A to G substitution at the -181 position influences the binding of various nuclear proteins resulting in a higher transcriptional activity [10]. A computational analysis also revealed that the -181G variant of MMP-7 gene causes a higher binding affinity of a cAMP-response element-binding protein and a stronger transcriptional activity [43]. It has also been found that subjects carrying the -181GG genotype have higher MMP-7 activity compared to those with the -181AA genotype [43]. This SNP has also been shown to correlate with the development of several kinds of tumors and chronic pancreatitis, as well as with coronary artery disease [44]. In this regard, Kesh and colleagues [43] showed increased expression of MMP-7 in cancer patients carrying the GG genotype. A significant association was also retrieved between the -181AA genotype and higher plasmatic levels of MMP7 among idiopathic pulmonary fibrosis patients [45]. A study by Kastelijn et al. [46] reported, however, that the -181 AA variant associates with lower concentration of MMP-7 in lung transplant recipients. To our best knowledge, there are no previous reports regarding the impact of MMP-7 -181A/G SNP on obesity and its anthropometric indicators, as well as on microvascular reactivity. From our findings, no significant relationships were found between the MMP-7 variant and the anthropometric parameters investigated (BMI, WC, and WHtR), which may suggest that this SNP does not affect body fat accumulation in the whole population. Interestingly, significant relationships among morbidly obese subjects were retrieved with the -181G allele, which correlate with a decreased microvascular response and a higher MMP-7 level. These results support those of Ress et al. [47] showing that increased MMP-7 levels in morbidly obese subjects might enhance adipocyte differentiation. As genetic impact on BMI may be higher in class III obesity, the -181A/G variant could, therefore, be a risk factor for the development of severe forms of obesity among Tunisians [48].

MMP-12 is a macrophage metalloelastase that has a role in ECM remodeling processes [6]. A previous report revealed an upregulation of MMP-12 in adipose tissue from obese subjects [49]. In a similar manner, increased levels of MMP-12 were found in mice with a diet-induced obesity and its expression colocalized in adipocytes and adipose tissue macrophages [50]. Maquoi and colleagues, [41] revealed, however, that a higher MMP-12 level during *in vitro* adipogenesis decreases adipocyte expansion by increasing endostatin levels and inhibiting adipose tissue vascularity. Up to now, several SNPs were reported in the MMP-12 locus. Among these, the -82A/G SNP was found to affect both AP-1 binding affinity and MMP-12 promoter activity [11]. A report by Motterle et al. [51] revealed that the -82A variant correlates with increased MMP-12 expression in atherosclerosis patients. This polymorphism has also been shown to correlate with several cardiovascular diseases. As an example, a study by Shimizu et al. [52] revealed a significant association between the -82G variant and coronary artery aneurysm. In a similar manner, the rs2276109 was associated with the extent of coronary artery disease and coronary atherosclerosis [53, 54]. With regard to obesity, no previous studies have determined the role of the -82A/G polymorphism on anthropometric traits and microvascular, as well as metabolic parameters. From our results, higher BMI values were associated with the -82G variant in the sampled population. Stratified analysis based on obesity status also revealed a significant association between the -82G allele and obesity, which reflects that the -82G allele is a risk factor for obesity among Tunisians. Significant relationships with the -82G allele were also obtained when nonmorbidly obese and morbidly obese subjects were considered, which reflects that this variant may be independently associated with obesity phenotype. We failed, however, to find significant relationships between this polymorphism and both MMP-12 levels and microvascular parameters in the sampled population, as well as in subgroups of healthy and obese subjects.

In the current report, when individuals were stratified by BMI, the MMP-3 45Glu (G) and MMP7 (-181G) variants were more frequent in individuals with severe forms of obesity. Although further studies are required, these results may highlight that the contribution of MMP-3 and MMP-7 SNPs in the development of the morbid phenotype can be substantially higher than in lower classes of BMI and could, therefore, be valuable markers to predict the risk for the development of extreme classes of obesity. Consistently, previous studies provided evidence of a weak population-related risk for common obesity for several genetic variants. As an example, Villalobos-Comparán et al. [55] found that the correlation between the G allele of PCSK1 gene (rs6232) and obesity was significant only in adult class III obesity. Also, León-Mimila and colleagues [56] revealed that the melanocortin 4 receptor variant (rs17782313) was associated with morbid obesity but not with class I/II obesity in a Mexican population. In another report, the FTO gene was a higher risk factor for obesity, especially in Portuguese subjects with class III obesity [57]. In a similar manner, the Ala55Val polymorphism (rs660339) of UCP2 gene was associated with severe forms of obesity in a Han Chinese population [58].

The limitations of this study should be considered. A first limitation is the small sample size of the screened population. Our findings need, therefore, to be replicated in a larger population or an independent severe obesity cohort. A second limitation is the lack of measures of MMP activities in serum, as well as in adipose tissue.

## 5. Conclusion

MMP-3 Lys45Glu (A/G), MMP-7 (-181A/G), and MMP-12 (-82A/G) variants were associated with obesity and its anthropometric indicators. The current findings also highlight a significant correlation between the MMP-3Lys45Glu (A/G) and MMP-7 (-181A/G) variants and severe forms of obesity among Tunisians. Our results also revealed significant alterations in microvascular reactivity in obese subjects. MMP variants may not, however, be determinant factors of endothelial dysfunction in the studied population.

## Supplementary Material

Supplementary file 1. Estimated haplotype frequencies of MMP-1, MMP-3, MMP-7 and MMP-12 polymorphisms in obese and non-obese subjects.

## Figures and Tables

**Table 1:** Details of PCR primer sequences and RFLP conditions.

Gene	Polymorphism	SNP	Primer sequence	PCR conditions	Restriction enzyme
MMP-1	-519A/G	rs1144393	F: 5′-CATGGTGCTATCGCAATAGGGT-3′ R: 5′-TGCTACAGGTTTCTCCACACAC-3′	Initial denaturation of 3 min at 94°C, followed by 35 cycles of 45 s at 94°C, 1 min at 60°C, 45 s at 72°C, and a final extension at 72°C for 10 min	KpnI
MMP-1	-1607 1G/2G	rs1799750	F: 5′-TCGTGAGAATGTCTTCCCATT-3′ R: 5′-TCTTGGATTGATTTGAGATAAG TGAAATC-3′	Initial denaturation of 2 min at 95°C, followed by 35 cycles of 45 s at 95°C, 1 min at 56°C, 45 s at 72°C, and a final extension at 72°C for 10 min	XmnI
MMP-3	Lys45Glu (A/G)	rs679620	F: 5′-CAACACCATAGCAGTAGCAGC-3′ R: 5′-CAGCCTCTCCTTCATACAGCC-3′	Initial denaturation of 3 min at 95°C, followed by 35 cycles of 1 min at 95°C, 45 s at 60°C, 45 s at 72°C, and a final extension at 72°C for 10 min	TaqI
MMP-7	-181A/G	rs11568818	F: 5′-TGGTACCATAATGTCCTGAATG-3′ R: 5′-TCGTTATTGGCAGGAAGCACACAATGAATT-3′	Initial denaturation of 3 min at 94°C, followed by 35 cycles of 45 s at 94°C, 1 min at 56°C, 45 s at 72°C, and a final extension at 72°C for 10 min	EcoRI
MMP-12	-82A/G	rs2276109	F: 5′-GAGATAGTCAAGGGATGATATCAGC-3′ R: 5′-AAGAGCTCCAGAAGCAGTGG-3′	Initial denaturation of 3 min at 94°C, followed by 35 cycles of 45 s at 94°C, 1 min at 60°C, 45 s at 72°C, and a final extension at 72°C for 10 min	PvuII

F: forward; MMP: matrix metalloproteinase; PCR: polymerase chain reaction; R: reverse; RLFP: restriction fragment length polymorphism; SNP: single nucleotide polymorphism.

**Table 2:** Anthropometric, biochemical, and microvascular parameters of the study subjects.

Parameters	All subjects	Obesity status		Classes of obesity		
		Nonobese (18.5 ≤ BMI < 25)	Obese (BMI ≥ 30)	Nonmorbidly obese		Morbidly obese
				Class I (30 ≤ BMI < 35)	Class II (35 ≤ BMI < 40)	Class III (BMI ≥ 40)
*Anthropometric measurements*						
BMI (kg/m^2^)	30 ± 0.44	22.87 ± 0.13	37.69 ± 0.5^∗∗^	32.16 ± 0.14	37.59 ± 0.21^**§**^	45.78 ± 0.75^#,ǂ^
WC (cm)	99.61 ± 1.2	79.93 ± 0.71	115.63 ± 1.06^∗∗^	105.69 ± 0.93	117.33 ± 1.37^**§**^	128.16 ± 1.6^#,ǂ^
HC (cm)	110.4 ± 1.06	95.07 ± 0.72	123.31 ± 1.12^∗∗^	113.82 ± 0.96	121.33 ± 1.65^**§**^	137.13 ± 1.6^#,ǂ^
WHR_ratio_	0.89 ± 0.005	0.84 ± 0.08	0.93 ± 0.006^∗∗^	0.92 ± 0.009	0.97 ± 0.01^**§**^	0.93 ± 0.11
WHtR_ratio_	0.6 ± 0.007	0.47 ± 0.004	0.7 ± 0.006^∗∗^	0.63 ± 0.005	0.71 ± 0.008^**§**^	0.8 ± 0.008 ^#,ǂ^
*Clinical measurements*						
SBP (mmHg)	121.6 ± 0.8	116.5 ± 0.58	126.4 ± 1.34^∗∗^	126.5 ± 1.85	128.6 ± 3.4	125.1 ± 2.3
DBP (mmHg)	79.4 ± 0.47	77 ± 0.43	81.8 ± 0.8^∗∗^	81.3 ± 1	82.4 ± 2	82.2 ± 1.35
MAP	79.05 ± 0.19	84.4 ± 1.81	74.73 ± 3.06	69.8 ± 0.47	74.64 ± 0.7	82 ± 4.67
*Biochemical parameters*						
hs-CRP (mg/dl)	3.7 ± 0.28	1.25 ± 0.18	5.79 ± 0.42^∗∗^	4.58 ± 0.52	5.45 ± 0.89	7.42 ± 0.82^#^
FG (mmol/l)	5.46 ± 0.07	5 ± 0.04	5.91 ± 0.12^∗∗^	5.69 ± 0.15	6.34 ± 0.34	5.98 ± 0.19
TG (mmol/l)	1.35 ± 0.04	1.15 ± 0.05	1.62 ± 0.06^∗∗^	1.55 ± 0.09	1.67 ± 0.11	1.69 ± 0.12
TC (mmol/l)	4.73 ± 0.05	4.45 ± 0.08	4.98 ± 0.07^∗∗^	4.96 ± 0.01	5.06 ± 0.13	4.97 ± 0.13
HDL-C (mmol/l)	1.18 ± 0.02	1.26 ± 0.03	1.11 ± 0.02^∗∗^	1.18 ± 0.03	1 ± 0.05^**§**^	1.07 ± 0.03
LDL-C (mmol/l)	2.9 ± 0.05	2.67 ± 0.07	3.16 ± 0.07^∗∗^	3.08 ± 0.11	3.29 ± 0.14	3.23 ± 0.14
ApoA1 (g/l)	1.67 ± 0.01	1.65 ± 0.02	1.68 ± 0.02	1.67 ± 0.03	1.7 ± 0.05	1.69 ± 0.03
ApoB (g/l)	0.9 ± 0.01	0.87 ± 0.02	0.93 ± 0.02	0.93 ± 0.03	0.96 ± 0.04	0.89 ± 0.03
*Microvascular function*						
FSBF_max_ (%)	595 ± 52.45	914.25 ± 77.43	299.8 ± 41.0^∗∗^	377.9 ± 68.38	226.71 ± 89.5	243.25 ± 56.73
FSBF_RHS_ (%)	1047.51 ± 65.78	946.02 ± 94.2	1139.6 ± 90.87	990.31 ± 138.7	1390 ± 228.2	1226.43 ± 137.02
AUC (PU^∗^Sec)	6623.8 ± 515.9	8168.6 ± 833.26	5307.84 ± 590.46^∗∗^	4863.54 ± 905.62	5424.45 ± 655.51	5723.01 ± 903.45
Basal CVC (PU/mmHg)	0.07 ± 0.007	0.09 ± 0.01	0.05 ± 0.003^∗∗^	0.06 ± 0.01	0.05 ± 0.01	0.06 ± 0.006
Peak ACh-CVC (PU/mmHg)	0.4 ± 0.03	0.48 ± 0.004	0.3 ± 0.03^∗∗^	0.26 ± 0.05	0.43 ± 0.18	0.32 ± 0.05
Peak RHS-CVC (PU/mmHg)	0.55 ± 0.02	0.59 ± 0.04	0.51 ± 0.03	0.67 ± 0.17	0.47 ± 0.06	0.53 ± 0.05
ΔACh-CVC (PU/mmHg)	0.32 ± 0.03	0.41 ± 0.04	0.25 ± 0.03^∗∗^	0.23 ± 0.05	0.41 ± 0.18	0.24 ± 0.05
ΔRHS-CVC (PU/mmHg)	0.46 ± 0.02	0.48 ± 0.0.04	0.44 ± 0.03	0.4 ± 0.06	0.43 ± 0.15	0.48 ± 0.05

^∗∗^ *P* < 0.001 (in nonobese versus obese subjects). ^§^*P* < 0.05 (in obese class I versus obese class II). ^#^*P* < 0.05 (in obese class I versus obese class III (morbid)). ^ǂ^*P* < 0.05 (in obese class II versus obese class III). ApoA1: apolipoprotein A1; ApoB: apolipoprotein B; AUC: area under the curve; BMI: body mass index; basal CVC: cutaneous vascular conductance; DBP: diastolic blood pressure; FG: fasting glucose; FSBF_max_: maximum forearm skin blood flow; FSBFRHS: forearm skin blood flow response to heating the skin; HC: hip circumference; HDL-C: high-density lipoprotein cholesterol; hs-CRP: high-sensitivity C reactive protein; LDL-C: low-density lipoprotein cholesterol; MAP: mean arterial pressure; PU: perfusion unit; SBP: systolic blood pressure; TC: total cholesterol; TG: triglycerides. Data are expressed as mean ± SD.

**Table 3:** Genotype distribution and allele frequencies of MMP-1 (-519A/G; -1607 1G/2G), MMP-3 Lys45Glu (A/G), MMP-7 (-181A/G), and MMP-12 (-82A/G) polymorphisms among nonobese and obese subjects.

SNP	Genotypes and alleles	Nonobese^£^ (*n* = 202, %)	Obese^£^ (*n* = 168, %)	Adjusted OR (95% CI)	*P*
*MMP-1 (-519A/G)*					
rs1144393	AA *n* (%)	106 (53)	65 (39.6)	1	
	AG *n* (%)	83 (41.5)	85 (51.8)	1.57 (1.02–2.41)	**0.03**
	GG *n* (%)	11 (5.5)	14 (8.6)	1.95 (0.84–4.55)	0.12
	AG+GG *n* (%)	94 (47)	99 (60.4)	1.61 (1.07–2.44)	**0.02**
	A *n* (%)	295 (73.75)	215 (65.55)	1	
	G *n* (%)	105 (26.25)	113 (34.45)	1.47 (1.07–2.03)	**0.02**
*MMP-1 (-1607 1G/2G)*					
rs1799750	1G1G *n* (%)	24 (12)	22 (13.4)	1	
	1G2G *n* (%)	75 (37.7)	62 (37.8)	0.88 (0.47–1.63)	0.68
	2G2G *n* (%)	100 (50.3)	80 (48.8)	0.9 (0.45–1.73)	0.73
	1G2G+2G2G *n* (%)	175 (88)	142 (86.6)	0.87 (0.45–1.67)	0.68
	1G *n* (%)	123 (30.9)	106 (32.32)	1	
	2G *n* (%)	275 (69.1)	222 (67.68)	0.93 (0.68–1.28)	0.7
*MMP-3 Lys45Glu (A/G)*					
rs679620	AA *n* (%)	69 (34.2)	33 (20.1)	1	
	AG *n* (%)	100 (49.5)	93 (56.7)	1.92 (1.16–3.18)	**0.01**
	GG *n* (%)	33 (16.3)	38 (23.2)	2.4 (1.30–4.5)	**0.006** ^∗^
	AG+GG *n* (%)	133 (65.8)	131 (79.9)	2.04 (1.26–3.3)	**0.004** ^∗^
	A *n* (%)	238 (58.91)	159 (48.48)	1	
	G *n* (%)	166 (41.09)	169 (51.52)	1.52 (1.13–2.04)	**0.004** ^∗^
*MMP-7 (-181A/G)*					
rs11568818	AA *n* (%)	53 (26.2)	38 (23)	1	
	AG *n* (%)	103 (51)	91 (55.2)	1.23 (0.74–2.03)	0.41
	GG *n* (%)	46 (22.8)	36 (21.8)	1.09 (0.60–1.99)	0.77
	AG+GG *n* (%)	149 (73.8)	127 (77)	1.19 (0.73–1.92)	0.48
	A *n* (%)	209 (51.73)	167 (50.61)	1	
	G *n* (%)	195 (48.27)	163 (49.39)	1.04 (0.78–1.4)	0.76
*MMP-12 (-82A/G)*					
rs2276109	AA *n* (%)	178 (88.6)	126 (76.4)	1	
	AG *n* (%)	23 (11.4)	38 (23)	2.33 (1.32–4.11)	**0.003** ^∗^
	GG *n* (%)	0	1 (0.6)	2.28	0.99
	AG+GG *n* (%)	23 (11.4)	39 (23.6)	2.39 (1.36–4.2)	**0.002** ^∗^
	A *n* (%)	379 (94.28)	290 (87.88)	1	
	G *n* (%)	23 (5.72)	40 (12.12)	2.27 (1.33–3.88)	**0.002** ^∗^

The chi-square (*χ*^2^) test was used for the determination of genotype distribution between nonobese and obese subjects. Adjustments for age and sex were performed by logistic regression analysis. CI: confidence interval; OR: odds ratio. ^∗^*P* values remained significant after the Bonferroni correction. ^£^The sum does not add up to the total because of a few missing genotypes in nonobese and obese subjects.

**Table 4:** Genotype distribution and allele frequencies of MMP-1 (-519A/G; -1607 1G/2G), MMP-3 Lys45Glu (A/G), MMP-7 (-181A/G), and MMP-12 (-82A/G) polymorphisms among nonobese, nonmorbidly obese, and morbidly obese.

SNP	Genotypes and alleles	Nonobese^£^ (*n* = 202, %)	Nonmorbidly obese^£^ (*n* = 108, %)	Adjusted OR (95% CI)	*P* ^a^	Morbidly obese^£^ (*n* = 60, %)	Adjusted OR (95% CI)	*P* ^b^
MMP-1 (-519A/G)								
(rs1144393)	AA *n* (%)	106 (53)	41 (38.4)	1		24 (42.1)	1	
	AG *n* (%)	83 (41.5)	59 (55.1)	1.5 (0.9–2.5)	0.12	26 (45.6)	1.38 (0.72–2.64)	0.33
	GG *n* (%)	11 (5.5)	7 (6.5)	1.6 (0.56–4.6)	0.38	7 (12.3)	3.01 (1.08–4.04)	0.05
	AG+GG *n* (%)	94 (47)	66 (61.6)	0.66 (0.4–1.1)	0.1	33 (57.9)	1.56 (0.84–2.9)	0.15
	A *n* (%)	295 (73.75)	141 (65.89)	1		74 (64.91)	1	
	G *n* (%)	105 (26.25)	73 (34.11)	1.45 (1.01-2.08)	0.06	40 (35.09)	1.51 (0.97–2.36)	0.06
MMP-1 (-1607 1G/2G)								
(rs1799750)	1G1G *n* (%)	24 (12)	16 (15)	1		6 (10.5)	1	
	1G2G *n* (%)	75 (37.7)	41 (38.3)	0.85 (0.39–1.86)	0.68	21 (36.8)	1.18 (0.41–3.41)	0.75
	2G2G *n* (%)	100 (50.3)	50 (46.7)	0.8 (0.37–1.72)	0.57	30 (52.7)	1.26 (0.45–3.52)	0.65
	1G2G+2G2G *n* (%)	175 (88)	91 (85)	0.82 (0.40–1.7)	0.6	51 (89.5)	1.23 (0.46–3.3)	0.67
	1G *n* (%)	123 (30.9)	73 (34.11)	1		33 (28.95)	1	
	2G *n* (%)	275 (69.1)	141 (65.89)	0.86 (0.60–1.23)	0.41	81 (71.05)	1.1 (0.70–1.73)	0.68
MMP-3 Lys45Glu (A/G)								
(rs679620)	AA *n* (%)	69 (34.2)	25 (23.5)	1		8 (13.8)	1	
	AG *n* (%)	100 (49.5)	59 (55.7)	1.51 (0.84–2.73)	0.16	34 (58.6)	2.83 (1.20–6.68)	**0.01**
	GG *n* (%)	33 (16.3)	22 (20.8)	1.8 (0.85–3.78)	0.12	16 (27.6)	4.93 (1.84–13.32)	**0.002** ^∗^
	AG+GG *n* (%)	133 (65.8)	81 (76.5)	1.58 (0.90–2.77)	0.1	50 (86.2)	3.31 (1.45–7.56)	**0.005** ^∗^
	A *n* (%)	238 (58.91)	109 (51.42)	1		50 (43.1)	1	
	G *n* (%)	166 (41.09)	103 (48.58)	1.35 (0.96–1.9)	0.07	66 (56.9)	1.9 (1.24–2.87)	**0.002** ^∗^
MMP-7 (-181A/G)								
(rs11568818)	AA *n* (%)	53 (26.2)	32 (30.2)	1		5 (8.5)	1	
	AG *n* (%)	103 (51)	56 (52.8)	0.99 (0.60–1.78)	0.99	36 (61)	3.68 (1.40–6.71)	**0.01**
	GG *n* (%)	46 (22.8)	18 (17)	0.65 (0.31–1.36)	0.65	18 (30.5)	3.4 (1.19–6.68)	**0.02**
	AG+GG *n* (%)	149 (73.8)	74 (69.8)	0.88 (0.51–1.54)	0.67	54 (91.5)	3.58 (1.40–9.14)	**0.008**
	A *n* (%)	209 (51.73)	120 (56.6)	1		46 (38.98)	1	
	G *n* (%)	195 (48.27)	92 (43.4)	0.82 (0.58–1.14)	0.25	72 (61.02)	1.67 (1.10–2.54)	**0.02**
MMP-12 (-82A/G)								
(rs2276109)	AA *n* (%)	178 (88.6)	82 (76.6)	1		43 (74.1)	1	
	AG *n* (%)	23 (11.4)	25 (23.4)	1.58 (0.90–2.77)	**0.01**	14 (24.1)	2.2 (1.21–3.97)	**0.01**
	GG *n* (%)	0	0		0.99	1 (1.8)		0.99
	AG+GG *n* (%)	23 (11.4)	25 (23.4)	1.58 (0.90–2.77)	**0.01**	15 (25.9)	2.6 (1.21–5.56)	**0.01**
	A *n* (%)	379 (94.28)	189 (88.32)	1		100 (86.21)	1	
	G *n* (%)	23 (5.72)	25 (11.68)	2.17 (1.20–3.94)	**0.008** ^∗^	16 (13.79)	2.63 (1.34–5.17)	**0.003** ^∗^

The chi-square (*χ*^2^) test was used for the determination of genotype distribution between nonobese and obese class I-II and between nonobese and obese class III. Adjustments for age and sex were performed by logistic regression analysis. *P*^a^ (nonobese versus obese class I-II) and *P*^b^ (nonobese versus obese class III (morbid)). CI: confidence interval; OR: odds ratio. ^∗^*P* value remained significant after the Bonferroni correction. ^£^The sum does not add up to the total because of a few missing genotypes.
