# Supplementary material for: Role of MMP-1 (-519A/G, -1607 1G/2G), MMP-3 (Lys45Glu), MMP-7 (-181A/G), and MMP-12 (-82A/G) Variants and Plasma MMP Levels on Obesity-Related Phenotypes and Microvascular Reactivity in a Tunisian Population
Source: Dis Markers. 2017 Nov 26;2017:6198526. doi: 10.1155/2017/6198526 (PMC5727656; doi:10.1155/2017/6198526)
Supplement: Supplementary file 1 — Estimated haplotype frequencies of MMP-1, MMP-3, MMP-7 and MMP-12 polymorphisms in obese and non-obese subjects. [file 6198526.f1.pdf]

Supplementary file 1. Estimated haplotype frequencies of MMP-1, MMP-3, MMP-7 and MMP-12 polymorphisms in obese and non-obese subjects.

Haplotypes					Haplotypes [n (%)]		OR (95 % CI)	P value
					Non-obese [n (%)]	obese [n (%)]		
$2G_{-1607}$	A_{-519}	A_{-181}	$A_{Lys45Glu}$	A_{-82}	0.19	0.11	1	---
$2G_{-1607}$	A_{-519}	G_{-181}	$G_{Lys45Glu}$	A_{-82}	0.13	0.14	1.42(0.38-5.28)	0.600
$2G_{-1607}$	A_{-519}	G_{-181}	$A_{Lys45Glu}$	A_{-82}	0.09	0.10	1.96(0.51-7.58)	0.330
$2G_{-1607}$	G_{-519}	G_{-181}	$A_{Lys45Glu}$	A_{-82}	0.03	0.09	1.05(0.25-8.53)	**0.031**
$1G_{-1607}$	A_{-519}	G_{-181}	$G_{Lys45Glu}$	A_{-82}	0.03	0.06	3.67(0.55-24.3)	0.180
$1G_{-1607}$	G_{-519}	G_{-181}	$A_{Lys45Glu}$	A_{-82}	0.02	0.06	1.71(0.15-18.90)	0.660
$1G_{-1607}$	G_{-519}	G_{-181}	$G_{Lys45Glu}$	A_{-82}	0.01	0.03	9.2(4.50-29.8)	**P<0.001***
Others								

Significant results (p values) are shown in bold. CI-confidence interval, OR-odds ratio. **P* values remained significant after Bonferroni Correction
